# Gas Sensors Based on Mechanically Exfoliated MoS_2_ Nanosheets for Room-Temperature NO_2_ Detection

**DOI:** 10.3390/s19092123

**Published:** 2019-05-08

**Authors:** Wenli Li, Yong Zhang, Xia Long, Juexian Cao, Xin Xin, Xiaoxiao Guan, Jinfeng Peng, Xuejun Zheng

**Affiliations:** 1 School of Physics and Optoelectronics, Xiangtan University, Xiangtan 411105, China; liwenli0917@163.com (W.L.); longxia0987@163.com (X.L.); xinx0907@163.com (X.X.); xiaox_guan@163.com (X.G.); 2 Hunan Institute of Advanced Sensing and Information Technology, Xiangtan University, Xiangtan 411105, China; 3 School of Mechanical Engineering, Xiangtan University, Xiangtan 411105, China; pengjinfeng1980@163.com (J.P.); zhengxuejun0419@163.com (X.Z.)

**Keywords:** MoS_2_ nanosheets, gas sensors, NO_2_ sensors, mechanical exfoliation

## Abstract

The unique properties of MoS_2_ nanosheets make them a promising candidate for high-performance room temperature gas detection. Herein, few-layer MoS_2_ nanosheets (FLMN) prepared via mechanical exfoliation are coated on a substrate with interdigital electrodes for room-temperature NO_2_ detection. Interestingly, compared with other NO_2_ gas sensors based on MoS_2_, FLMN gas sensors exhibit high responsivity for room-temperature NO_2_ detection, and NO_2_ is easily desorbed from the sensor surface with an ultrafast recovery behavior, with recovery times around 2 s. The high responsivity is related to the fact that the adsorbed NO_2_ can affect the electron states within the entire material, which is attributed to the very small thickness of the MoS_2_ nanosheets. First-principles calculations were carried out based on the density functional theory (DFT) to verify that the ultrafast recovery behavior arises from the weak van der Waals binding between NO_2_ and the MoS_2_ surface. Our work suggests that FLMN prepared via mechanical exfoliation have a great potential for fabricating high-performance NO_2_ gas sensors.

## 1. Introduction

In recent years, transition metal dichalcogenides (TMDs) have attracted much interest due to their unique layered structure, electronic and energy storage properties, which can be exploited in numerous devices such as sensors, field-effect transistors and supercapacitors [1,2,3,4]. MoS_2_, as the frontrunner in the TMDs family, has also been extensively investigated as a potential gas-sensing material because of its tunable band gap, large surface-to-volume ratio, and various active sites [5,6,7]. Gas-sensing properties are closely related to the size of the gas-sensing materials. According to reports, all electrons inside the gas sensing channel can be affected by the adsorbed gas when the grain size is smaller than two times the Debye length (so-called grain-size control). The space-charge layer then penetrates into the whole sensing channels, and the response is drastically promoted [8,9]. Structurally, MoS_2_ is a layered material in which S-Mo-S atoms are closely packed in a hexagonal arrangement, and each neighboring layer is connected by van der Waals forces [10]. Due to the strong intra-layer interactions and the relatively weak interactions between these layers, the synthesis of single or few-layer MoS_2_ nanosheets (FLMN) becomes possible by mechanical exfoliation from bulk MoS_2_ [11,12]. With this method which is the easiest and fastest way to produce the pristine, highly crystalline and atomic thickness layered materials [13], single or few-layer MoS_2_ nanosheets can be obtained without introducing too many defects. Compared with single-layer MoS_2_, few-layer MoS_2_ exhibits much higher electronic mobility due to lower Schottky barriers, which makes it more attractive for gas sensing [14,15,16]. Moreover, it is reported that the physical adsorption of gas molecules on MoS_2_ surface can overcome the shortcoming of difficult desorption from two-dimensional materials [17], which is beneficial to improve the recovery characteristics of gas sensors. Hence, the FLMN prepared via mechanical exfoliation show great potential in fabricating high-performance NO_2_ gas sensors. 

In this work, the FLMN prepared via mechanical exfoliation is transferred to an Al_2_O_3_ ceramic substrate with Ag-Pd interdigital electrodes. Exfoliated MoS_2_ nanosheets are interconnected among interdigital electrodes to form sensitive channels. The planar gas sensors based on FLMN show high responsivity to NO_2_ and ultrafast recovery behavior without heating unit. We hope that this work can provide a useful guideline for the application of two-dimensional (2D) MoS_2_ in high-performance gas sensors.

## 2. Experimental Details

**Figure 1:**
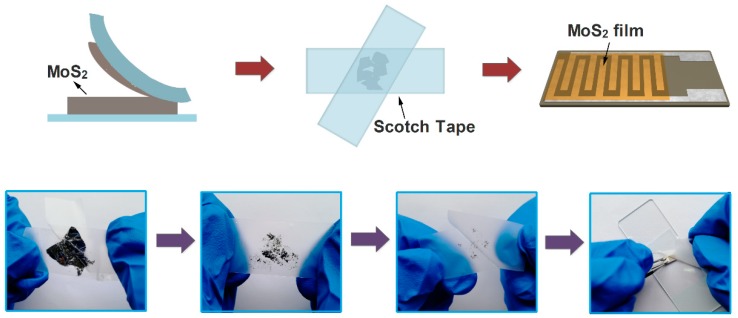
Schematic diagram of the preparation process for the mechanically exfoliated MoS_2_ nanosheets.

A typical mechanical exfoliation process is shown in Figure 1. The bulk MoS_2_ crystal and scotch tape were purchased from XFNANO Materials Tech Co. (Nanjing, China). First, a piece of scotch tape was adhered onto a bulk MoS_2_ crystal for about 5 s and then the scotch tape was removed carefully with as small angles as possible. Secondly, the scotch tape with the MoS_2_ flakes was folded and separated repeatedly many times to get thin MoS_2_ flakes, and a metallic luster can be clearly seen on the scotch tape surface. Then, the scotch tape with MoS_2_ thin flakes adhered tightly onto a clean Al_2_O_3_ ceramic substrate with Ag-Pd interdigital electrodes, and this adhesion state was maintained for 6 hours before the scotch tape was removed. Subsequently, the acetone was dripped onto the substrate to remove adhesive residue from the scotch tape. Finally, an FLMN gas sensor without the heating unit was obtained and used for further characterization.

The morphologies of exfoliated MoS_2_ nanosheets were observed by scanning electron microscope (SEM, Hitachi SU5000, Tokyo, Japan), transmission electron microscope (TEM, JEM-2100, JEOL, Tokyo, Japan) and atomic force microscope (AFM, Cypher S, Asylum Research, Oxford Instruments, Abingdon, UK). Molecular structure of exfoliated MoS_2_ nanosheets was evaluated by Raman spectra (Renishaw inVia, Renishaw, Gloucestershire, UK). The gas sensor surface was observed through a biological microscope (Eclipse-E200, Nikon, Tokyo, Japan). All the electrical measurements were carried out on a CGS-8 intelligent gas sensing analysis system (Beijing Elite Tech Co., Ltd, Beijing, China) at room temperature of 25 °C. The laboratory humidity is 55% relative humidity (RH), and the volume of our test chamber is 1000 mL. The NO_2_ gas sources of different concentrations (5 ppm, 10 ppm, 20 ppm, 50 ppm, 100 ppm, and 200 ppm, 21%vol O_2_ with 79%vol N_2_ as balanced gas) were bought from Dalian Special Gases co. LTD (Dalian, China), which had been calibrated by Fourier transform infrared spectrometer (spectrum 100, PerkinElmer, Waltham, MA, USA). The response of the gas sensors is defined as the ratio of the resistance of the sensors in tested gases (R_g_) to that in the air (R_0_). For oxidizing tested gases, that is response = R_g_/R_0_, while for the reducing tested gases, response = R_0_/R_g_ [18,19,20]. The time taken by a sensor to reach 90% of the total resistance change was defined as the response/recovery times [21].

## 3. Results and Discussion

**Figure 2:**
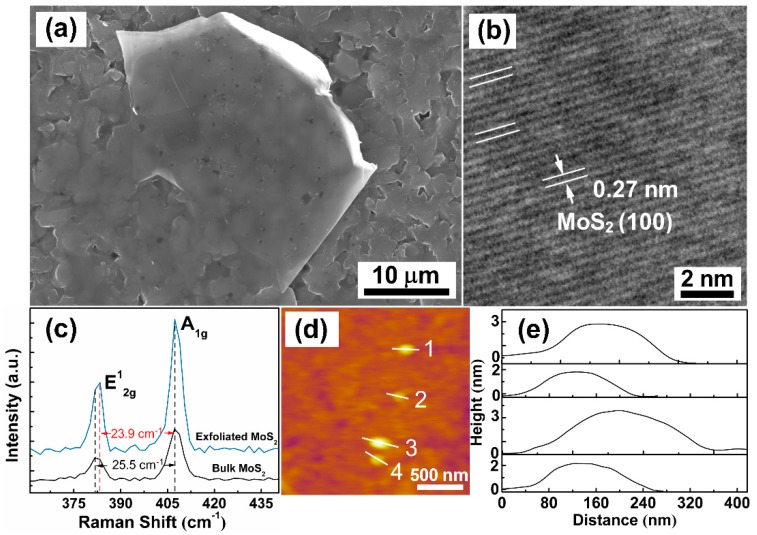
(**a**) SEM and (**b**) HRTEM images of MoS_2_ nanosheets. (**c**) Raman spectra of MoS_2_ nanosheets and bulk MoS_2_. (**d**) AFM image of MoS_2_ nanosheets. (**e**) Height profiles of the AFM image.

In order to further verify the few-layer microstructure of MoS_2_ nanosheets, SEM, TEM, Raman spectra and AFM images of MoS_2_ nanosheets are shown in Figure 2. From Figure 2a, a sheet with a smooth surface is observed on the substrate, and the sheet surface shows the same pattern as the substrate, which looks transparent. This phenomenon, that the fuzzy pattern of the substrate reveals on the sheet surface, can be mainly attributed to the fact that the thickness of the sheet is thin enough to allow the electron beam to penetrate through it at the acceleration voltage of 30 kV, resulting in the reception of signals from the substrates. A high-resolution TEM (HRTEM) image is shown in Figure 2b, and a lattice fringe spacing of 0.27 nm corresponding to the crystal planes (100) of MoS_2_. The ordered lattice arrangement of MoS_2_ indicates that MoS_2_ has good crystallinity [22]. In Figure 2c, Raman spectra of bulk MoS_2_ and exfoliated MoS_2_ nanosheets are shown as measured using a 532 nm laser at room temperature of 25 °C. Two characteristic vibration modes can be observed in the spectrum of bulk MoS_2_, the in-plane E^1^_2g_ mode at 381.8 cm^−1^ results from opposite vibration of two S atoms with respect to the Mo atom while the A_1g_ mode at 407.3 cm^−1^ is associated with the out-of-plane vibration of only S atoms in opposite directions [23,24]. Generally, the frequency difference (Δ) of the two dominant modes can be used to estimate the number of MoS_2_ layers [25].

Compared to the bulk MoS_2_, the E^1^_2g_ peak of MoS_2_ nanosheets shifts from 381.8 cm^−1^ to 383.4 cm^−1^, whereas the A_1g_ mode experiences almost zero shift. The value of Δ is 23.9 cm^−1^ for the exfoliated MoS_2_ nanosheets which consisted of about four-monolayer MoS_2_, agrees well with the reported results in the literature [23]. Figure 2d,e shows the AFM image and the corresponding quantitative AFM height profiles. The thicknesses of the randomly distributed MoS_2_ nanosheets are about 1.5 to 3.2 nm. With the thickness of a MoS_2_ monolayer of about 0.65 nm [26,27], this suggests that the as-prepared FLMN are composed of 2–5 monolayers MoS_2_.

**Figure 3:**
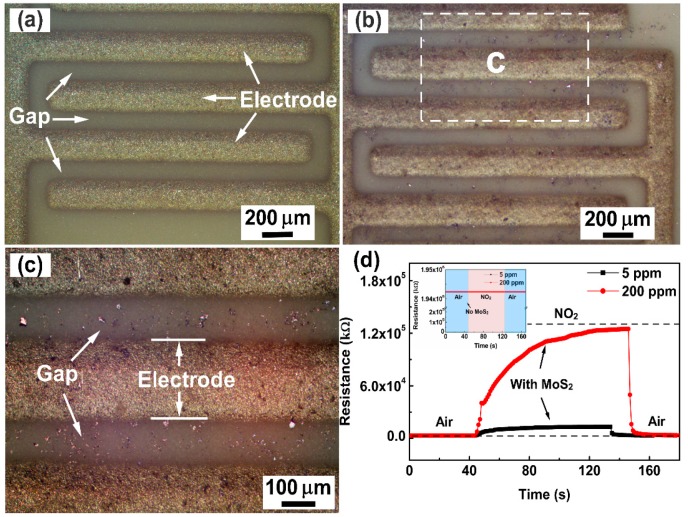
Optical images of the gas sensor (**a**) without FLMN and (**b**) with FLMN, and (**c**) optical images of the encircled region in FLMN gas sensor of Figure 3b with higher magnification. (**d**) Response/recovery characteristic curves of the gas sensor with and without FLMN to 5 ppm and 200 ppm NO_2_.

The optical images of the gas sensor without and with FLMN are displayed in Figure 3a–c. The blank Al_2_O_3_ ceramic substrate with Ag-Pd interdigital electrodes is clearly seen in Figure 3a, and the bare gap is quite clean without any materials. From Figure 3b,c, the FLMN with metallic luster are randomly dispersed on the Al_2_O_3_ ceramic substrate with interdigital electrodes, and the interlaced MoS_2_ nanosheets are bridged between the adjacent electrodes, which form the sensing channels on the gap. In order to support the issue that the sensing channels are composed of bridged MoS_2_ nanosheets, gas sensors with and without FLMN were exposed to 5 and 200 ppm NO_2_ at room temperature and their response and recovery behaviors are shown in Figure 3d. It can be seen that the resistance of the gas sensor coated with FLMN increases upon injection of NO_2_ and that it returns to its original value after removing the NO_2_. The higher the concentration, the larger the resistance change. The resistance responses of the uncoated gas sensor to 5 and 200 ppm NO_2_ are shown in the inset of Figure 3d. No matter whether the uncoated gas sensor is exposed to 5 ppm NO_2_, 200 ppm NO_2_ or air, there is no change in the resistance, and its resistance is much higher than the resistance of the gas sensor coated with FLMN. These results prove that bridging MoS_2_ nanosheets between adjacent electrodes form a conduction channel that reduces the resistance of the coated gas sensor below its resistance before coating. The observed NO_2_ response patterns can, therefore, be ascribed to the MoS_2_ nanosheets.

**Figure 4:**
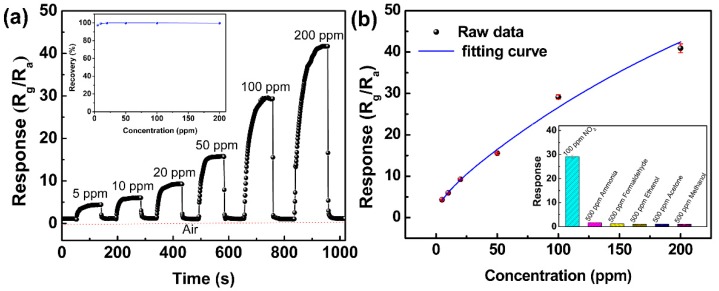
(**a**) Transient response characteristics at an NO_2_ concentration range of 5 to 200 ppm, and the inset shows the recovery rate of the FLMN gas sensor at different NO_2_ concentrations. (**b**) Index fitting curve of the response versus NO_2_ concentration and the inset shows the cross sensitivity of the FLMN gas sensor with regard to various target gases.

The FLMN gas sensor was repeatedly exposed to gas pulses with concentrations ranging from 5 to 200 ppm NO_2_, separated by periods of fresh air in between. The resulting transient response-recovery curve of the FLMN gas sensor is shown in Figure 4a. The resistance of the gas sensor gradually increases with the NO_2_ concentrations as it is exposed to NO_2_, implying that the N-type response behavior of MoS_2_ nanosheets is found in the detection of NO_2_. According to this figure, the response of the gas sensor continuously increases as the NO_2_ concentration is ramped up from 5 to 200 ppm at room temperature, and the responses are about 4.4, 6.1, 9.3, 15.8, 29.1 and 41.7 corresponding to 5, 10, 20, 50, 100 and 200 ppm NO_2_, respectively. After removing the NO_2_ from the gas sensor, the resistance of the gas sensor can return completely each time with almost no drift. The recovery behavior is very fast and recovery time constants are as short as 2–4 s. As the MoS_2_ gas sensors reported in the previous works often fail to recover [28,29,30], the degree of recovery is an important indicator of the quality of the gas sensor. Herein, the recovery characteristic of the MoS_2_ gas sensor is investigated by calculating the recovery rate, defined as follows [31,32].

(1) $$\mathrm{Recovery}\;\mathrm{rate}\;\left(\%\right)=\frac{R_{g}-R_{r}}{R_{g}-R_{0}}\times 100.\;$$

Here, R_0_ and R_g_ are the resistances of the gas sensor before and after exposure to the target gas, and R_r_ is the stable resistances after putting the gas sensor back to air. As shown in the inset of Figure 4a, the FLMN gas sensor shows an outstanding recovery rate greater than 97%, which implies good recovery behavior for the FLMN gas sensor to detect NO_2_ gas.

In order to illustrate the contrast between our gas sensor and the state of the art, the performance of our gas sensor is compared to other reported MoS_2_ gas sensor in Table 1. The gas sensor based on FLMN presents a very high response of 4.4 and a very fast recovery time of ~2 s at 5 ppm NO_2_ gas, which does not require any heating unit to realize the detection of NO_2_ at room temperature. 

As far as we know, our gas sensor features the fastest recovery time and the highest response for the detection of NO_2_ at room temperature. In order to illustrate the data reliability, error bars have been calculated by the standard deviation formula [40], and the response versus NO_2_ concentration index fitting curve with error bars is shown in Figure 4b. It exhibits small deviations for the FLMN gas sensor, indicating the data are reliable in the whole concentration range from 5 to 200 ppm. Based on the least squares method [41], the fitting equation of the response Y and NO_2_ concentration X can be represented as Y = 81.68 − 77.26 × e^(−X/295.29)^ − 2.44 × e^(−X/10.48)^, and the regression coefficient R^2^ is 0.965 at the concentration range from 5 to 200 ppm. The response curve shows optimal linear dependence in range of 5 to 100 ppm and then sign of slight saturation behavior at the NO_2_ concentration larger than 100 ppm. From the inset of Figure 4b, the FLMN gas sensor shows a high response to NO_2_ at room temperature while only minimal responses toward other gases such as ammonia, formaldehyde, ethanol, acetone and methanol, which is of an excellent cross sensitivity toward NO_2_. 

**Figure 5:**
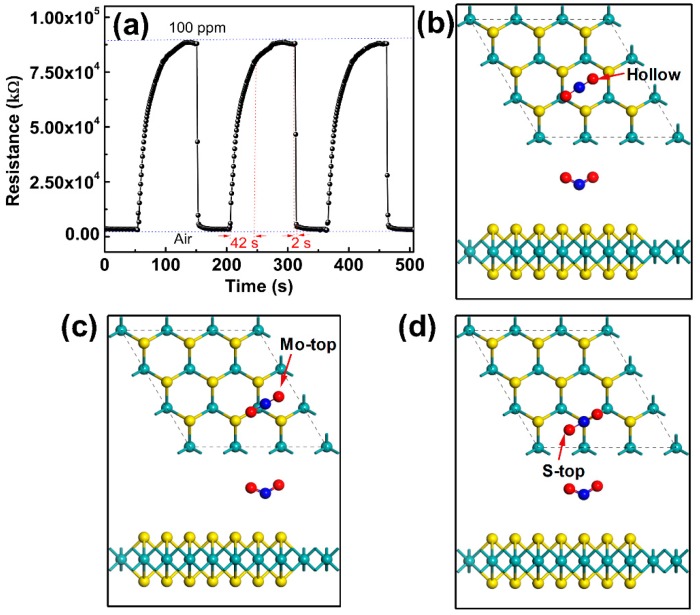
(**a**) Repeatability and reversibility of the FLMN gas sensor at 100 ppm NO_2_ concentration. (**b**−**d**) Three adsorption configurations of NO_2_ molecules on MoS_2_ surface.

In order to evaluate the repeatability and reversibility, the FLMN gas sensor is continuously placed into and removed from NO_2_ of the same concentration, and Figure 5a shows the response and recovery curves for three cycles when the gas sensors alternately change between air and 100 ppm NO_2_. Generally, the target gas is difficult to desorb completely from gas sensor surface without any stimulation of external field such as thermal field [36], optical field [42], etc., resulting in the long recovery time of gas sensor and the large drifting baseline at room temperature [31,32]. The above phenomenon is mainly caused by the chemical adsorption formed on the surface of sensing materials which makes it difficult for gas molecules to desorption [43]. In our work, for each cycle, the response of the gas sensor is 29 (the resistance changes from about 3 MΩ to 88 MΩ in the case of NO_2_ adsorption) and the response/recovery times are 42/2 s as shown in Figure 5a. It is worth mentioning that the recovery curve can return quickly to the baseline for each time with almost no drift, namely, the gaseous NO_2_ can be completely desorbed from the gas sensor without any extra stimulus like optical or thermal sources. The results illustrate that our FLMN gas sensors are superior in terms of repeatability and reversibility compared to other gas sensors working at room temperature. Interestingly, and contrary to many reported works [20,32,44,45,46], the recovery time of our sensors is far shorter than response time. The reason for this abnormal behavior is attributed to the fact that physical adsorption of gases is dominant on the mechanically exfoliated FLMN surface which is reported to have few defects [47]. While chemical adsorption mainly takes place on the other reported gas sensors due to the formation of defects on the surface of MoS_2_ synthesized by the wet chemical method [48,49]. In order to verify the physical adsorption behavior of NO_2_ on the MoS_2_ surface mentioned above, the parameters related to the adsorption configuration, such as the adsorption energy, the distance between NO_2_ and MoS_2_, the bond lengths of gas molecules, etc., are calculated based on density-functional theory (DFT). All DFT calculations were performed as implemented in the Vienna ab-initio simulation package (VASP) [50,51], and the exchange-correlation potential is treated with the Perdew-Burke-Eznerh of generalized-gradient approximation (PBE-GGA) [52,53]. The projector augmented wave (PAW) method is used to describe the electron–ion interaction [54]. For the structural relaxations and energy calculations, we employ the D2 method of Grimme (DFT-D2), which includes van der Waals (vdW) interactions [52,55]. All calculations are performed with a 3 × 3 × 1 supercell of MoS_2_ containing 27 atoms, and the cut off energy for plane-wave expansion is 400 eV. The Brillouin zone is sampled with a grid of 9 × 9 × 1 conducted by the Monkhorst-Pack special k-point scheme [56]. For geometry optimization, all the internal coordinates are fully relaxed until the Hellmann-Feynman forces are less than 0.005 eV/Å. The three adsorption configurations of NO_2_ molecules on MoS_2_ surface, including the NO_2_ adsorbed on the hollow, Mo-top and S-top sites of MoS_2_, are shown in Figure 5b–d, and Table 2 gives the adsorption parameters for three adsorption configurations including total energy (E_tot_), adsorption energy (E_ad_), the distance of adsorbed NO_2_ to the MoS_2_ monolayer (*d*_zN-S_) and the bond length of gas molecules (*l*_N-O_). The adsorption energy of gas molecules on MoS_2_ surface is calculated as E_ad_ = E_tot_ − E_MoS_2__ − E_Gas_, where E_tot_ is the total energy of MoS_2_ with a molecule absorbed, E_MoS_2__ and E_Gas_ are the energies of the pristine MoS_2_ single layer and isolated gas molecule. From Table 2, the total adsorption energies of the three adsorption configurations are almost the same, indicating that three adsorption configurations are all possible for the adsorption of NO_2_ on MoS_2_ surface. 

The E_ad_ of NO_2_ in the three adsorption configurations is very small, which the maximum E_ad_ (NO_2_ adsorbed on hollow) is only 0.05 eV, and the distances of adsorbed NO_2_ to the MoS_2_ surface are large in all three adsorption configurations (*d*_zN-S_ are all greater than 3 Å). The small E_ad_ and the large *d*_zN-S_ indicate that no chemical bond is formed between NO_2_ and MoS_2_ on MoS_2_ surface. Moreover, no matter at which adsorption site NO_2_ is adsorbed, the *l*_N-O_ of NO_2_ gas molecules is approximately 1.218 Å, which *l*_N-O_ is almost unchanged compared with *l*_N-O_ (*l*~1.213 Å) of the free NO_2_ gas molecule. This further illustrates that the NO_2_ gas is not chemically adsorbed on the MoS_2_ surface, because the bond length will be greatly affected by the gas chemically adsorbed on the surface of the material [57,58]. Therefore, from the calculation results of E_ad_, *d*_zN-S_ and *l*_N-O_, it is concluded that the NO_2_ gas is adsorbed on the MoS_2_ surface by a weak van der Waals interaction, i.e. the physical adsorption is the main factor for the adsorption of NO_2_ on MoS_2_ surface. 

The response of gas sensing materials against target gas is mainly dependent on the electronic interaction between gases and materials, which occurs mainly on the surface of materials, i.e., it is a surface-controlled process [59]. As only a certain thickness of material surface can interact with the gas, only a certain depth of electron depletion layer can be formed with an order of 2–100 nm when the gas adsorbs on the surface of materials, which is usually called the Debye length (L) [8,9,60]. When the crystallite size (D) is much larger than 2L, grain-boundary contacts display higher resistance and govern the electric gas sensitivity of the chain (grain-boundary control) [59]. When D decreases to come closer to 2L, the necks become the most resistant, controlling the gas sensitivity (neck control) [61]. Finally, when D is smaller than 2L, each constituent grain is fully depleted of conduction electrons as a whole. In this situation, the resistance of grains dominates the whole resistance of the chain and the gas sensitivity, in this case, is controlled by grains themselves (grain control) [9]. The reported results mentioned above illustrate that the gas sensing properties are closely related to the size of gas sensing materials. 

**Figure 6:**
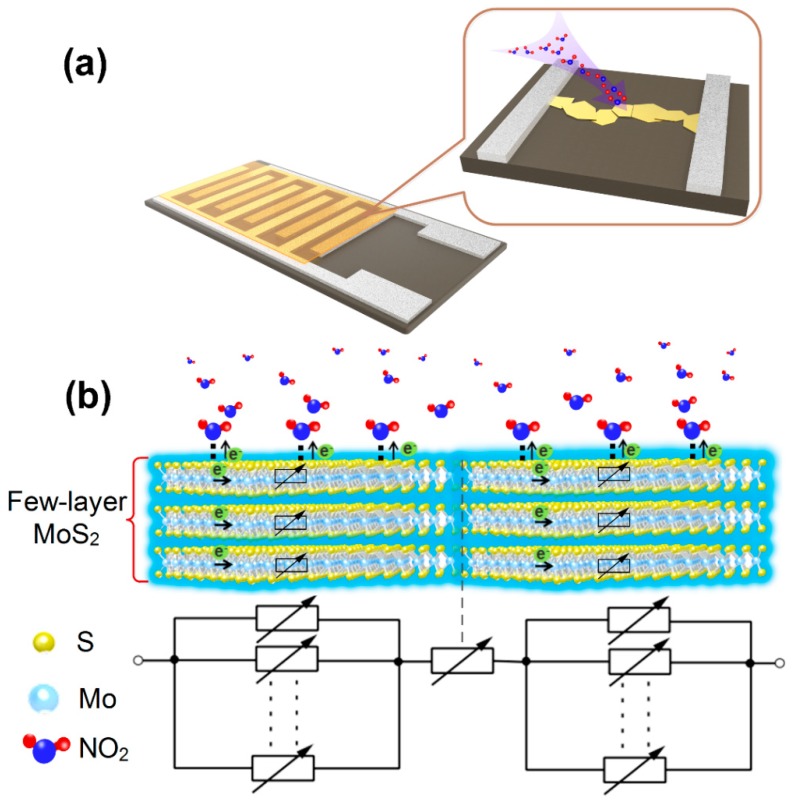
(**a**) Schematic diagram of the FLMN gas sensor structure. (**b**) Schematic diagram of NO_2_ gas sensing mechanism and equivalent circuit for the FLMN.

Figure 6 shows the schematic diagram of the gas sensing mechanism and equivalent circuit, and the exfoliated FLMNs are bridged randomly between the adjacent electrodes, which form the sensing channels on the gap. In our work, the gas sensor based on FLMN exhibits a higher response than other reported MoS_2_ gas sensors which have been shown in Table 1, and their high responsivity can be attributed to the following two reasons. First, the thickness of MoS_2_ is about 1.5–3.2 nm in Figure 6b, which belongs to the type of grain control, so the electrons of all over the sensing channels (including the bridging contact and the FLMN itself) can be affected by the adsorbed NO_2_, and the space-charge layer then penetrates into the whole sensing channels, which leads to a sharp decrease in conductivity and great improvement of the response [8,9]. As shown in the equivalent circuit diagram, all the electronic transport paths through the FLMN are controlled by NO_2_ gas, so the total resistance of FLMN gas sensor can change greatly when cycling it between air and NO_2_. As we have shown in the schematic of Figure 6b, the main reason why FLMN have a large response is that the NO_2_ gas controls all the conduction channels of carriers in the material, and the thickness of MoS_2_ is an important factor determining its gas sensing properties. Secondly, the gas molecules are generally confined to adsorb on the active sites of materials in the case of chemical adsorption, which limits the number of gas molecules adsorbed on the surface of the material [62,63]. By means of physical adsorption and a small amount of chemical adsorption, NO_2_ gas molecules can easily diffuse to the whole surface of the material, leading to the increase of the adsorption quantity of gas molecules on the materials surface [63]. When gas molecules are physically adsorbed the material surface, the electrostatic attraction between the material and gas causes the transformation of electrons from the surface of the material to form a dipole moment [47,64,65]. As the gas concentration increases, the continuous transfer of electrons will increase the resistance of materials, and the increase of dipole moment further promotes physical adsorption of gas molecules. Therefore, the resistance of material increases with the increase in gas concentration. In addition, there may be a few chemisorptions of NO_2_ gas molecules on the mechanically exfoliated FLMN due to the existence of few defects on the surface of MoS_2_ [66]. Here, chemisorption of NO_2_ gas molecules on FLMN does not affect the fast desorption behavior, and the main reason is that Brunauer–Emmett–Teller (BET) water layers formed on FLMN surface may dissolve the chemisorbed NO_2_ which can promote the rapid desorption of chemisorbed NO_2_ [67]. 

## 4. Conclusions

In summary, an FLMN gas sensor via a facile way (mechanical exfoliation) was demonstrated to have excellent performance, which enables overcoming the limitations of 2D TMDs gas sensors such as low response and poor recovery. Through the comparison with the state of the art, the performances, including high response against NO_2_ at room temperature and the quick and complete recovery behaviors (the recovery time of 2 s, the recovery rate greater than 97%), are confirmed in this work. Based on density-functional theory (DFT), the calculation shows that the excellent performances at room temperature are mainly attributed to the physical adsorption of NO_2_ on FLMN surface and size effect from extremely thin thickness of FLMN. Thus, an FLMN gas sensor via mechanical exfoliation can resolve the low NO_2_-sensing performance issues in terms of response and recovery, and potentially open up a new avenue for gas sensing applications. 

## Figures and Tables

**Table 1:** Compared gas-sensing performances of few-layer MoS_2_ nanosheets with previous works based on different MoS_2_ nanostructures toward NO_2_.

Materials	NO_2_ (ppm)	T (°C)	Responsivity	T_rec_	Ref.
Few-layer MoS_2_ nanosheets	5 ppm	RT	4.4	2 s	This work
Multilayer MoS_2_	5 ppm	RT	1.153	…	[33]
MoS_2_ film	5 ppm	RT	1.092	>5 min	[34]
MoS_2_ nanosheets	5 ppm	RT	3.8	…	[35]
MoS_2_ nanowires	5 ppm	60	1.819	172 s	[36]
Mixed MoS_2_ flakes	10 ppm	RT	1.116	…	[37]
Mixed MoS_2_ flakes	10 ppm	125	1.085	19.6 s	[37]
Plane MoS_2_	50 ppm	RT	5.5	…	[28]
MoS_2_ nanoflowers	40 ppm	RT	~1.23	…	[29]
Vertically aligned MoS_2_	50 ppm	RT	~1.483	…	[30]
Vertically aligned MoS_2_	50 ppm	100	~1.28	>30 s	[30]
MoS_2_ hollow sphere	100 ppm	150	1.403	225 s	[38]
2D MoS_2_	500 ppm	RT	4	8 min	[39]

Note: RT = room temperature. T_rec_ = recovery time.

**Table 2:** Calculated adsorption parameters of NO_2_ molecule in its three adsorption configurations.

Configuration	E_tot_ in eV	E_ad_ in eV	*d*_zN-S_ in Å	*l*_N-O_ in Å
Hollow	−214.250	−0.050	3.128	1.218
Mo-top	−214.221	−0.021	3.120	1.217
S-top	−214.227	−0.027	3.124	1.218

## References

[1] Cho B., Yoon J., Lim S.K., Kim A.R., Choi S.-Y., Kim D.-H., Lee K.H., Lee B.H., Ko H.C., Hahm M.G. (2015). Metal decoration effects on the gas-sensing properties of 2D hybrid-structures on flexible substrates. Sensors.

[2] Huang Y., Guo J., Kang Y., Ai Y., Li C.M. (2015). Two dimensional atomically thin MoS_2_ nanosheets and their sensing applications. Nanoscale.

[3] Bhimanapati G.R., Lin Z., Meunier V., Jung Y., Das S., Cha J., Xiao D., Son Y., Strano M.S., Cooper V.R. (2015). Recent advances in two-dimensional materials beyond graphene. ACS Nano.

[4] Shang M., Du C., Huang H., Mao J., Liu P., Song W. (2018). Direct electrochemical growth of amorphous molybdenum sulfide nanosheets on Ni foam for high-performance supercapacitors. J. Colloid Interface Sci..

[5] Su S., Lv W., Zhang T., Tan Q., Zhang W., Xiong J. (2018). A MoS_2_ Nanoflakes-Based LC Wireless Passive Humidity Sensor. Sensors.

[6] Park S.Y., Kim Y.H., Lee S.Y., Sohn W., Lee J.E., Kim D.H., Shim Y.-S., Kwon K.C., Choi K.S., Yoo H.J. (2018). Highly selective and sensitive chemoresistive humidity sensors based on rGO/MoS_2_ van der Waals composites. J. Mater. Chem. A.

[7] Cho S.-Y., Kim S.J., Lee Y., Kim J.-S., Jung W.-B., Yoo H.-W., Kim J., Jung H.-T. (2015). Highly enhanced gas adsorption properties in vertically aligned MoS_2_ layers. ACS Nano.

[8] Yamazoe N. (1991). New approaches for improving semiconductor gas sensors. Sens. Actuators B Chem..

[9] Seal S., Shukla S. (2002). Nanocrystalline SnO gas sensors in view of surface reactions and modifications. JOM.

[10] Pujari R.B., Lokhande A.C., Shelke A.R., Kim J.H., Lokhande C.D. (2017). Chemically deposited nano grain composed MoS_2_ thin films for supercapacitor application. J. Colloid Interface Sci..

[11] Mak K.F., Lee C., Hone J., Shan J., Heinz T.F. (2010). Atomically thin MoS_2_: A new direct-gap semiconductor. Phys. Rev. Lett..

[12] Novoselov K.S., Jiang D., Schedin F., Booth T.J., Khotkevich V.V., Morozov S.V., Geim A.K. (2005). Two-dimensional atomic crystals. Proc. Natl. Acad. Sci. USA.

[13] Li M.-Y., Chen C.-H., Shi Y., Li L.-J. (2016). Heterostructures based on two-dimensional layered materials and their potential applications. Mater. Today.

[14] Perera M.M., Lin M.-W., Chuang H.-J., Chamlagain B.P., Wang C., Tan X., Cheng M.M.-C., Tománek D., Zhou Z. (2013). Improved carrier mobility in few-layer MoS_2_ field-effect transistors with ionic-liquid gating. ACS Nano.

[15] Liu H., Neal A.T., Ye P.D. (2012). Channel Length Scaling of MoS_2_ MOSFETs. ACS Nano.

[16] Das S., Chen H.-Y., Penumatcha A.V., Appenzeller J. (2012). High-performance Multilayer MoS_2_ Transistors with Scandium Contacts. Nano Lett..

[17] Zhao J., Li N., Yu H., Wei Z., Liao M., Chen P., Wang S., Shi D., Sun Q., Zhang G. (2017). Highly sensitive MoS_2_ humidity sensors array for noncontact sensation. Adv. Mater..

[18] Xu H., Ju D., Li W., Gong H., Zhang J., Wang J., Cao B. (2016). Low-working-temperature, fast-response-speed NO_2_ sensor with nanoporous-SnO_2_/polyaniline double-layered film. Sens. Actuators B Chem..

[19] Xiao Y., Yang Q., Wang Z., Zhang R., Gao Y., Sun P., Lu G. (2016). Improvement of NO_2_ gas sensing performance based on discoid tin oxide modified by reduced graphene oxide. Sens. Actuators B Chem..

[20] Cho J.-H., Yu J.-B., Kim J.-S., Sohn S.-O., Lee D.-D., Huh J.-S. (2005). Sensing behaviors of polypyrrole sensor under humidity condition. Sens. Actuators B Chem..

[21] Feng J., Sun X., Wu C., Peng L., Lin C., Hu S., Yang J., Xie Y. (2011). Metallic few-layered VS_2_ ultrathin nanosheets: High two-dimensional conductivity for in-plane supercapacitors. J. Am. Chem. Soc..

[22] Li H., Zhang Q., Yap C.C.R., Tay B.K., Edwin T.H.T., Olivier A., Baillargeat D. (2012). From bulk to monolayer MoS_2_: Evolution of raman scattering. Adv. Funct. Mater..

[23] Lin H., Wang J., Luo Q., Peng H., Luo C., Qi R., Huang R., Travas-Sejdic J., Duan C.-G. (2017). Rapid and highly efficient chemical exfoliation of layered MoS_2_ and WS_2_. J. Alloy. Compd..

[24] Lee C., Yan H., Brus L.E., Heinz T.F., Hone J., Ryu S. (2010). Anomalous lattice vibrations of single-and few-layer MoS_2_. ACS Nano.

[25] Radisavljevic B., Radenovic A., Brivio J., Giacometti V., Kis A. (2011). Single-layer MoS_2_ transistors. Nat. Nanotechnol..

[26] Lee H.S., Min S.-W., Park M.K., Lee Y.T., Jeon P.J., Kim J.H., Ryu S., Im S. (2012). MoS_2_ nanosheets for top-gate nonvolatile memory transistor channel. Small.

[27] Shim Y.-S., Kwon K.C., Suh J.M., Choi K.S., Song Y.G., Sohn W., Choi S., Hong K., Jeon J.-M., Hong S.-P. (2018). Synthesis of Numerous Edge Sites in MoS_2_ via SiO_2_ Nanorods Platform for Highly Sensitive Gas Sensor. ACS Appl. Mater. Interfaces.

[28] Kanaujiya N., Anupam, Golimar K., Pandey P.C., Jyoti, Varma G.D. (2018). Investigating NO_2_ gas sensing behavior of flower-like MoS_2_ and rGO based nano-composite. AIP Conf. Proc..

[29] Kumar R., Kulriya P.K., Mishra M., Singh F., Gupta G., Kumar M. (2018). Highly selective and reversible NO_2_ gas sensor using vertically aligned MoS_2_ flake networks. Nanotechnology.

[30] Chatterjee A.P., Mitra P., Mukhopadhyay A.K. (1999). Chemically deposited zinc oxide thin film gas sensor. J. Mater. Sci..

[31] Ko K.Y., Song J.-G., Kim Y., Choi T., Shin S., Lee C.W., Lee K., Koo J., Lee H., Kim J. (2016). Improvement of gas-sensing performance of large-area tungsten disulfide nanosheets by surface functionalization. ACS Nano.

[32] Kumar R., Goel N., Kumar M. (2017). UV-Activated MoS_2_ Based Fast and Reversible NO_2_ Sensor at Room Temperature. ACS Sens..

[33] Xu T., Pei Y., Liu Y., Wu D., Shi Z., Xu J., Tian Y., Li X. (2017). High-response NO_2_ resistive gas sensor based on bilayer MoS_2_ grown by a new two-step chemical vapor deposition method. J. Alloy. Compd..

[34] Han Y., Huang D., Ma Y., He G., Hu J., Zhang J., Hu N., Su Y., Zhou Z., Zhang Y. (2018). Design of Heteronanostructures on MoS_2_ Nanosheets to Boost NO_2_ Room Temperature Sensing. ACS Appl. Mater. Interfaces.

[35] Kumar R., Goel N., Kumar M. (2018). High-performance NO_2_ sensor using MoS_2_ nanowires network. Appl. Phys. Lett..

[36] Agrawal A.V., Kumar R., Venkatesan S., Zakhidov A., Yang G., Bao J., Kumar M., Kumar M. (2018). Photoactivated Mixed In-Plane and Edge-Enriched p-Type MoS_2_ Flake-Based NO_2_ Sensor Working at Room Temperature. ACS Sens..

[37] Li Y., Song Z., Li Y., Chen S., Li S., Li Y., Wang H., Wang Z. (2019). Hierarchical hollow MoS_2_ microspheres as materials for conductometric NO_2_ gas sensors. Sens. Actuators B Chem..

[38] Zhao Y., Song J.-G., Ryu G.H., Ko K.Y., Woo W.J., Kim Y., Kim D., Lim J.H., Lee S., Lee Z. (2018). Low-temperature synthesis of 2D MoS_2_ on a plastic substrate for a flexible gas sensor. Nanoscale.

[39] Zhang H.G., Han X.J., Yao B.F., Li G.X. (2013). Study on the effect of engine operation parameters on cyclic combustion variations and correlation coefficient between the pressure-related parameters of a CNG engine. Appl. Energy.

[40] Li J., Hou C., Huo D., Yang M., Fa H.B., Yang P. (2014). Development of a colorimetric sensor array for the discrimination of aldehydes. Sens. Actuators B Chem..

[41] Zhou Y., Gao C., Guo Y. (2018). UV assisted ultrasensitive trace NO_2_ gas sensing based on few-layer MoS_2_ nanosheet-ZnO nanowire heterojunctions at room temperature. J. Mater. Chem. A.

[42] Lee G., Yang G., Cho A., Han J.W., Kim J. (2016). Defect-engineered graphene chemical sensors with ultrahigh sensitivity. Phys. Chem. Chem. Phys..

[43] Wang Z., Zhang Y., Liu S., Zhang T. (2016). Preparation of Ag nanoparticles-SnO_2_ nanoparticles-reduced graphene oxide hybrids and their application for detection of NO_2_ at room temperature. Sens. Actuators B Chem..

[44] Gu D., Li X., Zhao Y., Wang J. (2017). Enhanced NO_2_ sensing of SnO_2_/SnS_2_ heterojunction based sensor. Sens. Actuators B Chem..

[45] Randeniya L.K., Shi H., Barnard A.S., Fang J., Martin P.J., Ostrikov K. (2013). Harnessing the Influence of Reactive Edges and Defects of Graphene Substrates for Achieving Complete Cycle of Room-Temperature Molecular Sensing. Small.

[46] Ricciardella F., Vollebregt S., Polichetti T., Miscuglio M., Alfano B., Miglietta M.L., Massera E., Francia G.D., Sarro P.M. (2017). Effects of graphene defects on gas sensing properties towards NO_2_ detection. Nanoscale.

[47] Huo N., Yang S., Wei Z., Li S.S., Xia J.B., Li J. (2014). Photoresponsive and gas sensing field-effect transistors based on multilayer WS_2_ nanoflakes. Sci. Rep..

[48] Kang J., Ikram M., Zhao Y., Zhang J., Rehman A.U., Gong L., Shi K. (2017). Three-dimensional flower-like Mg(OH)_2_@MoS_2_ nanocomposite: Fabrication, characterization and high-performance sensing properties for NOx at room temperature. New J. Chem..

[49] Xie J., Zhang H., Li S., Wang R., Sun X., Zhou M., Zhou J., Xie Y. (2013). Defect-rich MoS_2_ ultrathin nanosheets with additional active edge sites for enhanced electrocatalytic hydrogen evolution. Adv. Mater..

[50] Kresse G., Furthmüller J. (1996). Efficient iterative schemes for ab initio total-energy calculations using a plane-wave basis set. Phys. Rev. B.

[51] Kresse G., Furthmüller J. (1996). Efficiency of ab-initio total energy calculations for metals and semiconductors using a plane-wave basis set. Comp. Mater. Sci..

[52] Kohn W., Sham L.J. (1965). Self-consistent equations including exchange and correlation effects. Phys. Rev..

[53] Perdew J.P., Burke K., Ernzerhof M. (1996). Generalized gradient approximation made simple. Phys. Rev. Lett..

[54] Bucko T., Hafner J., Lebegue S., Angyan J.G. (2010). Improved description of the structure of molecular and layered crystals: Ab initio DFT calculations with van der Waals corrections. J. Phys. Chem. A.

[55] Monkhorst H.J., Pack J.D. (1976). Special points for Brillouin-zone integrations. Phys. Rev. B.

[56] Zhou M., Lu Y.-H., Cai Y.-Q., Zhang C., Feng Y.-P. (2011). Adsorption of gas molecules on transition metal embedded graphene: A search for high-performance graphene-based catalysts and gas sensors. Nanotechnology.

[57] Gao G., Park S.H., Kang H.S. (2009). A first principles study of NO_2_ chemisorption on silicon carbide nanotubes. Chem. Phys..

[58] Liu B., Chen L., Liu G., Abbas A.N., Fathi M., Zhou C. (2014). High-performance chemical sensing using Schottky-contacted chemical vapor deposition grown monolayer MoS_2_ transistors. ACS Nano.

[59] Tang W., Wang J. (2016). Enhanced gas sensing mechanisms of metal oxide heterojunction gas sensors. Acta Phys.-Chim. Sin..

[60] Sharma S., Madou M. (2012). A new approach to gas sensing with nanotechnology. Phil. Trans. R. Soc. A.

[61] Xu C., Tamaki J., Miura N., Yamazoe N. (1991). Grain size effects on gas sensitivity of porous SnO_2_-based elements. Sens. Actuators B Chem..

[62] Cho Y., Sohn A., Kim S., Kim D.-W., Cho B., Hahm M.G., Kim D.-H. (2016). Influences of gas adsorption and Au nanoparticles on the electrical properties of CVD-grown MoS_2_ thin films. ACS Appl. Mater. Interfaces.

[63] Qi L., Wang Y., Shen L., Wu Y. (2016). Chemisorption-induced n-doping of MoS_2_ by oxygen. Appl. Phys. Lett..

[64] Yang J.H., Ji J.L., Li L., Wei S.H. (2014). Hydrogen Chemisorption and Physisorption on the Two-Dimensional TiC Sheet Surface. Acta Phys.-Chim. Sin..

[65] Zhao S., Xue J., Kang W. (2014). Gas adsorption on MoS_2_ monolayer from first-principles calculations. Chem. Phys. Lett..

[66] Fang H., Chuang S., Chang T.C., Takei K., Takahashi T., Javey A. (2012). High-performance single layered WSe_2_ p-FETs with chemically doped contacts. Nano Lett..

[67] Maier K., Helwig A., Müller G., Hille P., Eickhoff M. (2015). Effect of water vapor and surface morphology on the low temperature response of metal oxide semiconductor gas sensors. Materials.

